# The Relationship Between Defense Mechanisms and Attachment as Measured by Observer-Rated Methods in a Sample of Depressed Patients: A Pilot Study

**DOI:** 10.3389/fpsyg.2021.648503

**Published:** 2021-09-27

**Authors:** Vera Békés, Katie Aafjes-van Doorn, Daniel Spina, Alessandro Talia, Claire J. Starrs, J. Christopher Perry

**Affiliations:** ^1^Ferkauf Graduate School of Psychology, Yeshiva University, New York, NY, United States; ^2^Department of Clinical Psychology, Pennsylvania State University, University Park, PA, United States; ^3^Institute for Psychosocial Prevention, University Hospital Heidelberg, University of Heidelberg, Heidelberg, Germany; ^4^Department of Psychology, SUNY Potsdam, Potsdam, NY, United States; ^5^Department of Psychiatry, Canada Institute of Community and Family Psychiatry, Jewish General Hospital, McGill University, Montreal, QC, Canada

**Keywords:** defense mechanisms, attachment, depression, observer-rated, patient attachment coding system

## Abstract

Despite many theoretical and clinical writings, the theorized connection between defense mechanisms and adult attachment in depressed patients has received little empirical attention. This is the first study to examine patients’ defense mechanisms in relation to their attachment in a clinical sample of depressed patients and also the first to use observer-rated measures for assessing both defense mechanisms and attachment. In this pilot study, we aimed to investigate the relationship between patients’ attachment and their use of defense mechanisms in psychotherapy sessions, as well as patterns of change over treatment. We conducted a secondary analysis of data from a randomized controlled trial of 30 patients receiving psychotherapy for major depression. Session transcripts were previously coded for defense mechanisms using the Defense Mechanisms Rating Scales, and depression severity data were collected by the clinician-rated HRSD-17 and the self-report BDI-II. Patients’ attachment was assessed in two transcripts, one in an early session and a second in a late session, using the novel observer-rated Patient Attachment Coding System. In contrast with expectations, in the early phase of therapy, preoccupied attachment-related characteristics were significantly positively related to overall defensive functioning and negatively related to Depressive immature defenses. In the late phase of treatment, preoccupied attachment-related characteristics were negatively correlated with Non-depressive immature defenses. Moreover, as expected, early-phase defense use was related to late phase attachment; specifically, early neurotic and immature Depressive and Non-depressive defenses predicted an increase in avoidant, whereas immature Non-depressive defenses predicted a decrease in preoccupied attachment-related characteristics over the course of treatment, after controlling for early attachment effects. The results imply a longitudinal relationship between defenses and change in attachment-related characteristics over the course of treatment in a depressed sample and warrant further research about the relationship between defenses and attachment during psychotherapy.

## Introduction

Patients’ attachment-related differences and defense mechanisms are the two main aspects of personality functioning and are thought to be important predictors of symptom severity and psychotherapy outcome (Blatt and Levy, 2003; Perry, 2014; Dagan et al., 2018; Perry et al., 2020). Despite increasing interest in the topic over the past few years, there is still little empirical research conducted on the associations between defense mechanisms and patient’s attachment, especially in depressed patients. In this study, we sought to address this important gap in the literature by empirically examining the relationship between patients’ attachment-related characteristics and their use of defense mechanisms in treatment sessions conducted as part of a previous RCT for depression.

Defense mechanisms can be defined as automatic reactions to internal and external stressors or conflicts aimed at warding off negative emotional experiences. They are thought to underlie a wide range of healthy and psychopathological phenomena, including depression (Perry, 2014). The use of defense mechanisms in any given situation is mostly out of the individual’s awareness; however, the type of defense mechanism used can lead to considerable differences in mental health and interpersonal functioning (Vaillant, 2020).

Defense mechanisms can be categorized hierarchically, based on their general level of adaptiveness (Perry, 1990; Perry and Bond, 2017). Of the tripartite defense categories, *mature* defense mechanisms are deemed the most adaptive strategies to maximize gratification and allow relatively good conscious awareness of feelings, ideas, and their behavior-related consequences. Though all defense mechanisms are thought to protect the individual from anxiety, mature defenses do not threaten interpersonal relationships or distort reality in order to do so. The intermediate level of *neurotic* defense mechanisms functions to keep distressing thought content out of awareness, also with minimal reality distortion. In contrast, the low level, mostly maladaptive *immature* defenses act through strong reality distortion or detachment from reality (Perry and Bond, 2017) and are associated with mental health problems and lower interpersonal functioning, characteristic of severe mood and anxiety disorders (Trower and Chadwick, 1995; Calati et al., 2010; Perry and Bond, 2012; Berney et al., 2014; Ciocca et al., 2017).

Relevant to patients who suffer from depression, the immature defense category can be further subdivided into Depressive and Non-depressive Defenses. Depressive defenses have been empirically associated with depression, whereas Non-depressive defenses were negatively associated with depression (Høglend and Perry, 1998). In depressed patients, the use of immature defenses has been found to decrease by the end of treatment, whereas neurotic and mature defenses remain unchanged (e.g., Mullen et al., 1999). Moreover, within immature defenses, the subgroup of Depressive defense mechanisms is linked to decreases in depression symptomatology specifically (Perry et al., 2020).

Attachment theory (Bowlby, 1969) offers a cogent framework for understanding the development and treatment of psychopathologies such as depression (Cummings and Cicchetti, 1990; Williams and Riskind, 2004; Dykas and Cassidy, 2011; Lakey and Orehek, 2011; Hames et al., 2013). There appears to be an overrepresentation of patients with insecure attachment in clinical populations in general and in clinically depressed samples in particular, compared with non-clinical samples (Bakermans-Kranenburg and van Ijzendoorn, 2009; for a recent meta-analysis see Dagan et al., 2018). Similarly, individuals with insecure attachments have been shown to experience higher levels of depression than securely attached individuals (Fonagy et al., 1996; Borelli et al., 2010; Ivarsson et al., 2010).

John Bowlby developed his theory of attachment partly to explain why some of his patients appeared to eschew intimacy and defend against experiencing emotions, with calamitous consequences for their social adaptation (Duschinsky, 2020). Bowlby posited that individual differences in early relationships with one’s primary caregivers are carried forward and shape relationships with others (e.g., peers and romantic partners; Bowlby, 1988; Roisman, 2006; Feeney, 2008; Holland and Roisman, 2010; Groh et al., 2014).

Following Bowlby’s innovative theorizing, a host of studies have confirmed that early differences in attachment relationships later impact cognitive and affective processing of expectations about closeness and support from others. Beginning in the sixties, attachment researchers established that differences in parental sensitivity and responsiveness give rise to distinct infant tendencies to establish proximity with the caregiver, which in turn seem to be underpinned by differing expectations concerning caregiver availability (Ainsworth et al., 1978). In particular, Ainsworth and colleagues proposed that infants seek proximity with their caregiver in one of three ways: *secure*, involving actively seeking proximity if they generally expect the caregiver to be available when they are distressed; *avoidant*, if they do not hold such an expectation, they seem to defensively inhibit their search for physical proximity; and *resistant* (or ambivalent), if they expect the caregiver to be unpredictable or inconsistent leading to constantly monitoring their proximity to the caregiver even when he or she is within reach.

Later work showed that these infant differences are robustly predicted by parent’s attachment representations, as assessed in a semi-structured interview, the Adult Attachment Interview (AAI; Main et al., 1985). Namely, parents of secure infants in the AAI appear to openly access their own representations and memories of their relationships with their parents and are termed “secure-autonomous.” Parents of avoidant infants seem to shift their attention away from discussing attachment relationships and stressful episodes and are termed “dismissing,” while parents of resistant infants appear to focus excessively on such topics and are termed “preoccupied.”

According to one popular view, whereas secure attachment is related to an unbiased way of processing affectively laden information, with little need to use reality-distorting defense mechanisms (Cramer and Kelly, 2010; Dykas and Cassidy, 2011), insecure attachment reflects defensive responses to negative emotions, threats to separation, or distress more generally (Ein-Dor et al., 2016). In this view, attachment is seen as an adaptation strategy to a given environment (Luyten et al., 2021).

Certain defense mechanisms are prominent in the interpersonal patterns that convey the effect of attachment insecurity on psychological distress, such as depression. For example, dismissing attachment classifications seem to be associated with denying one’s own weaknesses and those of one’s attachment figures (Main et al., 2002). Conversely, preoccupied attachment may be associated with hyperactivating the expression of distress and maintaining a consistent focus on negative emotions, which may work to gain and maintain others’ proximity – at least in the short term.

Indeed, attachment theory can be understood as a two-person theory of conflict and defense. It emphasizes the coping or defensive processes required to deal with fearful arousal within the context of attachment relationships. In Bowlby’s view, defensive exclusion occurs when attachment-related information is kept out of awareness to prevent the painful effect associated with attachment system activation when no perceived comfort from attachment figures (real or representational) is available (Bowlby, 1980). In contrast to an intrapsychic theory of defense, attachment theory locates the ontogeny of defenses in an intersubjective field. The development of defensive styles is theorized to occur at the interface between a child’s fearful arousal and the subsequent responses of important attachment figures. More specifically, the infant-caregiver interactions that occur around distress and comfort result in defensive adaptations, in the form of defense mechanisms (Lyons-Ruth, 2003). In other words, in relation to adult attachment patterns, defenses are conceptualized as the mechanism that modulates the attachment system in order to reduce distressing feelings associated with negative expectancies, both at the intrapersonal and interpersonal levels (Kobak and Bosmans, 2019), and as such are directly related to emotion dysregulation (Malik et al., 2015).

Despite many theoretical and clinical writings, this hypothesized connection between attachment and defense mechanisms has received little empirical attention. The few existing empirical studies generally suggest that insecure attachment is typically associated with an increased use of immature defense mechanisms (e.g., Prunas et al., 2019) and that this overreliance on immature defenses leaves insecurely attached individuals particularly vulnerable to psychopathology, such as depression (e.g., Laczkovics et al., 2018; Ciocca et al., 2020). Up until now, however, empirical studies investigating the association between attachment and defenses have been conducted in non-clinical samples (Ciocca et al., 2020) rather than clinical or treatment samples.

Previous studies on the relationship between attachment and defense mechanisms have been further limited by their reliance on self-report questionnaires. Self-report measures may be more biased (when compared to observer-based measures) when aiming to identify processes that are predominantly unconscious, such as attachment and defenses. Whereas preliminary evidence shows that self-report and observer-rated defense ratings may align (Di Giuseppe et al., 2020), it is increasingly well-agreed that self-report measures of attachment (for example, the Experience of Close Relationships Scale; Brennan et al., 1998) and observer-rated measures of attachment (such as the AAI) do not cohere empirically and may in fact capture different constructs (Roisman, 2006; Strauss et al., 2015).

In the current study, we sought to address this gap in the literature by examining the association between attachment and defense mechanisms in patients undergoing psychotherapy for depression, using a novel observer-rated method for assessing attachment, the Patient Attachment Coding System (PACS; Talia et al., 2017), in addition to the well-established observer-rated DMRS for defenses. The PACS was initially developed in an effort to find verbal markers that would distinguish the discourse of patients who had been independently classified as secure, dismissing, or preoccupied on the AAI (Talia et al., 2014, 2017, 2019b). This work led to distinct identifying markers that can be reliably scored in any session of psychotherapy transcribed verbatim, regardless of the therapeutic orientation (Talia et al., 2014). Because the PACS markers occur regardless of whether patients speak about attachments or other topics that they find distressful, Talia and his colleagues have described them first and foremost as capturing differing ways in which patients collaborate with the therapist, rather than defenses (Talia et al., 2019a).

### Aims

Given the importance of attachment security and defense mechanisms in the development of psychopathology, such as depression (Høglend and Perry, 1998; Martin-Joy et al., 2017) and their general importance in treatment formulations (e.g., Fonagy, 2001; Eagle, 2013), it is important to better understand the relationship between these two processes. Thus, the overall aim of our study was to investigate the relationship between patients’ attachment and their use of defense mechanisms in psychotherapy for depression, as well as any patterns of change over time. Of note, in contrast with previous studies, where attachment style was assessed as a predictor of defense use, in this present pilot study, we aimed to explore the role of defense mechanism in predicting changes in in-session attachment-related characteristics over treatment. Specifically, we explored the following two research questions:

1. What is the relationship between depressed patients’ in-session attachment-related characteristics and their defense mechanisms? We hypothesized that patients with secure attachment would exhibit higher overall defensive functioning, would use more mature defenses, and less immature defenses, in both the early and late sessions. Conversely, we also expected that patients with insecure attachment, specifically avoidant and preoccupied patterns, would use more immature defenses, in particular more Depressive defenses.
2. Does patients’ defensive functioning in the early session predict their attachment security in the late phase of treatment? We expected that patients’ overall defensive functioning, and amount of mature or immature defense use, early in treatment would predict attachment-related characteristics in the late phase of treatment. More specifically, within this clinically depressed sample, we expected that lower-level defenses, such as Immature, and especially, early Depressive Immature defense use would predict insecure (avoidant and preoccupied) attachment-related characteristics in the late phase of treatment.

## Materials and Methods

### Treatment Trial

This study reports on secondary analyses of existing treatment data collected as part of a previously conducted randomized controlled treatment trial (RCT) of 30 patients undergoing treatment for major depression (see Perry et al., 2021 for a detailed description of the RCT). Inclusion criteria in the study were having acute recurrent major depression and a 17 or higher score on the Hamilton Depression Rating Scale; exclusion criteria included psychotic or bipolar type I disorders, substance use or dependence serious enough to interfere with therapy, and an effective response to antidepressant medications, if tried, in the past 4weeks.

Nineteen patients (63%) were female, and mean age was 41years (*SD*=12.43). As part of the RCT, patients were randomly assigned to either cognitive behavior psychotherapy (CBT; *n*=13), supportive psychotherapy (ST; *n*=7), or psychodynamic psychotherapy (PDT; *n*=10). On average, the CBT treatments consisted of 21.00 (*SD*=10.44) sessions over 14months (range=2.75–21.75) and the ST consisted of 17.00 (*SD*=9.04) sessions over 14months (6.5–27.5), whereas the PDT treatments were longer and consisted of an average of 62.7 (*SD*=23.43) sessions over 21months (range=7.5–24.5). Depressive symptoms were assessed at baseline and at the end of treatment. Baseline depression scores on the BDI-II (*M*=23.34, *SD*=6.97) and HRSD-17 (*M*=17.48, *SD*=6.10) significantly correlated (*r*=0.48, *p*<0.01), and both significantly decreased by termination [*t*(27)=5.63, *p*<0.001 and *t*(27)=4.22, *p*<0.001, respectively]. As a part of the original RCT, the treatment sessions were audio-recorded and transcribed and coded for individual defense mechanisms, hierarchically organized into subsequent defense categories. For further details on the trial and the participants, please see Perry et al. (2021).

### Measures

#### Existing Measurements

##### Depression

The clinician-rated Hamilton Depression Rating Scale (HRSD-17; Hamilton, 1960) was used to assess depression levels pre-and post-treatment. The HRSD-17 is a 17-item semi-structured interview, which assesses depression on a 5-point Likert scale, ranging from 0 to 4. The HRSD-17 has demonstrated good internal consistency in previous studies with a mean alpha of 0.79 across studies, in our report Cronbach’s alpha=0.83.

The self-report Beck Depression Inventory II (BDI-II; Beck, et al., 1996) was also administered pre-and post-treatment. The BDI-II is a widely used 21-item measure of Depressive symptoms experienced during the previous week, using a four-point Likert scale. Internal consistency of the BDI-II has been reported to be good in several studies, for example, a Cronbach alpha of 0.90 has been reported (Storch et al., 2004). Cronbach’s alpha for BDI-II was 0.96 in the present report.

##### Defense Mechanisms

The observer-rated Defense Mechanism Rating Scales (DMRS; Perry, 1990) was used to assess defense mechanisms in session transcripts in the early and late treatment phases. The DMRS identifies 30 individual defenses (Perry, 1990) as they occur in the text. The individual defense mechanisms are hierarchically arranged into three categories: Mature, Neurotic, and Immature defenses, and the Immature category can be further subdivided into Depressive and Non-depressive immature defenses. In addition to the tripartite categories, a score for overall defensive functioning (ODF) is calculated by summing the weighted average of each defense based on its defense level. The ODF can range between 1 and 7, with higher scores indicating more adaptive defensive functioning. Inter-rater reliability of the three defense categories, the Depressive and Non-depressive defenses, and the ODF have been shown to be satisfactory (Perry, 2014).

#### Novel Observer-Rated Method

##### Attachment

For this secondary analysis, the Patient Attachment Coding System (PACS; Talia et al., 2014) was used to assess patients’ attachment. The PACS is a transcript-based measure that yields classifications of patients’ attachment based on a single therapy session transcribed verbatim in any treatment modality, regardless of the stage of treatment and of therapist’s activity. Recent work in attachment-informed psychotherapy research (Talia et al., 2017) has shown that patients’ discourse style during psychotherapy reliably predicts their independently obtained attachment classification on the AAI. PACS attachment security has been found to predict greater in-session mentalizing (Talia et al., 2017), greater resolution of relational ruptures in psychotherapy (Miller-Bottome et al., 2018), and patient-therapist physiological synchrony (Kleinbub et al., 2020). The PACS has also been shown to predict patients’ AAI classification even when applied to post-treatment interviews rather than therapy sessions (Talia et al., 2019b).

When coding with the PACS, the coder assesses the frequency and intensity of 40 different discourse markers as they occur in a transcript, which are grouped into five main scales used to assign a final main attachment classification to the patient: Proximity seeking, Exploring, and Contact maintaining which are associated with Secure attachment; Avoidance which is associated with Avoidant attachment; and Resistance which is associated with Preoccupied attachment. A sixth scale, Balance, is used as a global score of security which encompasses the five main PACS scales. As such, although a person may exhibit predominantly secure attachment characteristics, they may also exhibit some avoidant and resistant markers.

In this study, we report on the scores on the three PACS scales reflecting attachment-related characteristics, including secure attachment (Balance scale), avoidant attachment (Avoidance scale), and preoccupied attachment (Resistance scale). In order to avoid multiple testing of related variables, we used Balance as a proxy for attachment security (and did not include the three secure scales). The rater assigns a rating from 1 to 7 in 0.5 increments based on both the frequency and intensity of the markers of each subscale identified in the transcript. More specifically, the Balance score reflects the degree of attachment security exhibited by the patient including the open expression of emotions in the present, communication of feeling and needs in the therapeutic relationship, autonomous reflections, and positive emotions. The Avoidance scale assesses the level of evasion of inquiries into the patient’s positive and negative experience and the level of minimization or deferment of any mental state previously conveyed (e.g., the patient affirms that he or she has no right to complain; chuckles about his or her own distress). The Resistance scale captures discourse markers that enlist the therapist’s agreement with the patient’s views or otherwise restrict the therapist’s capacity to disagree, for example, by being vague or excessively detailed. In order to assign an overall attachment classification (Secure, Avoidant, or Preoccupied) for the patient, a proportional index of balance, avoidant, and resistant characteristics is calculated (for a more detailed description of the PACS, see Talia et al., 2017).

### Procedures

In order to become reliable PACS coders, four clinical psychology doctoral students completed a one-week comprehensive training workshop in the use of the PACS taught by the developer (A.T.) and attended weekly reliability consensus meetings on practice transcripts for 3months following the training workshop. When their ICC with the developer of the PACS reached 0.80 or above, the students started coding the session transcripts for the study. Session transcripts were randomly assigned across the four raters. Throughout the coding, the raters received ongoing intensive supervision from the developer of the PACS. Inter-rater reliability was calculated on 29 (50%) out of 58 coded sessions, and the ICC between the developer and the coders was 0.85. From the available session transcripts already coded on the DMRS, two sessions per treatment were coded with the PACS, one session from the early phase of treatment (the second session) and a session at the late phase of treatment (the penultimate session), altogether resulting in a sample of 60 PACS coded sessions, reflecting 30 treatments.

### Data Analysis

In the reported analyses, the total sample of 30 treatments was used. Two patients were dropped out during treatment; therefore, the cross-sectional analysis at the early phase was based on *n*=30, whereas the analyses at the late phase of treatment and the change across treatment included *n*=28. The use of an existing data set and observer ratings meant that there were no missing attachment or defense scores. To compare initial attachment and defense scores across the three treatment arms, we conducted one-way ANOVA. The small number of patients in each treatment modality only allowed us to conduct pilot comparisons and to report effect sizes and not values of *p*.

The attachment and defense variables were not normally distributed (skewness and kurtosis more than twice the standard error). Both at the early and late phases, attachment scores on the Balance scale were significantly positively skewed, due to the high prevalence of insecure patients in the sample (*n*=21). Therefore, non-parametric tests of defenses and attachment were used in subsequent analyses. Wilcoxon signed-rank test was used to compare attachment and defenses in the early and late phases of the treatments. A paired samples *t* test was used to compare self-rated and observer-rated depression scores at pre-and post-treatment. Spearman’s rho correlations were used to analyze the relationship between variables on the DMRS and the PACS. Linear regression analysis was used to examine whether early-phase defensive functioning predicted late-phase attachment. For checking the assumptions for the regression models, we confirmed that the data contained approximately normally distributed errors with equal variance and met the assumptions of homogeneity of variance and linearity. Two-tailed tests of significance were applied throughout. Given the exploratory nature of the examinations and the relatively low power, we did not apply a correction for multiple correlations. All statistical analyses were conducted using SPSS 24.0.

## Results

### Patient Attachment and Defenses Early in Treatment

In the early sessions, the majority of the 30 patients were classified on the PACS as Preoccupied (*n*=15). Nine patients were classified as Secure and six as Avoidant. Regarding the scales, the average rating on the PACS Balance scale suggested that overall the patients in this sample were relatively insecurely attached (*M*=2.93; *SD*=1.4) at baseline, a score which is significantly lower than in other mixed outpatients’ samples [*M*=3.7, *SD*=1.3, *t*(188)=2.79, *p*<0.01; Talia et al., 2017]. Moreover, these depressed patients also scored higher on the PACS Resistance scale (*M*=4.20; *SD*=2.47), indicating that their attachment was significantly more preoccupied than is generally seen in outpatient samples [*M*=3.3, *SD*=2.00, *t*(188)=2.18, *p*<0.05], whereas the PACS Avoidance scale (*M*=2.79; *SD*=1.77) was in line with previous findings [*M*=2.8, *SD*=1.60, *t*(188)=0.00, *p*=ns; Talia et al., 2017].

Average overall defensive functioning (*M*=4.88; SD=0.57) early in treatment fell into the level usually associated with acute depression or personality disorders and was comparable to other mixed outpatient groups reported in the literature [*M*=4.62, *SD*=0.27, *t(49)*=1.93, *p*=ns.; Perry and Henry, 2004]. Table 1 shows the means, standard deviations, and significant changes in the relevant variables.

**Table 1:** Wilcoxon signed-rank tests comparing Beginning and Late-Phase Defense and Attachment Variables (*N*=28).

	Early Phase		Late Phase			
	Mean (*SD*)	Range	Mean (*SD*)	Range	*z*	*p*
**Attachment-related characteristics**						
PACS Balance	2.93 (1.40)	1.5–6.0	2.36 (0.98)	1.0–4.5	−1.50	0.134
PACS Avoidance	2.79 (1.77)	1.0–7.0	2.82 (1.77)	1.0–6.5	−0.16	0.871
PACS Resistance	4.20 (2.47)	1.0–7.0	4.68 (2.56)	1.0–7.0	−0.57	0.573
**Defense Mechanisms^a^**						
DMRS ODF	4.88 (0.57)	3.1–5.8	5.08 (0.49)	4.1–6.0	−1.34	0.179
DMRS Mature	0.17 (0.11)	0.0–0.4	0.17 (0.11)	0.0–0.4	−0.42	0.674
DMRS Neurotic	0.54 (0.16)	0.3–0.8	0.59 (0.17)	0.2–0.9	−1.35	0.178
DMRS Immature	0.28 (0.13)	0.1–0.6	0.23 (0.13)	0.0–0.5	−1.91	0.056
Immature: Depressive	0.18 (0.11)	0.0–0.6	0.15 (0.11)	0.0–0.5	−1.42	0.156
Immature: Non-depressive	0.10 (0.07)	0.0–0.2	0.08 (0.04)	0.0–0.3	−1.23	0.219

a Defense scores were obtained from the original RCT, see Perry et al. (2020), in this same journal issue.

Early-phase PACS and defense variables differed in the three treatment arms. Pilot comparison using Eta-squared showed that variance in early treatment PACS variables across the three treatment arms was Balance *η*^2^ =0.010, Avoidance *η*^2^ =0.158, and Resistance *η*^2^ =0.154; and variance based on the treatment arms in early-phase defense variables ranged between Neurotic defenses *η*^2^ =0.035 and ODF *η*^2^ =0.108.

### Research Question 1: Relationship Between the Patients’ Attachment-Related Characteristics and Their Use of Defense Mechanisms

Spearman’s rho correlations were used to test the relationship between in-session attachment-related characteristics (PACS Balance, PACS Avoidance, and PACS Resistance) at both early and late phases of treatment and patients’ use of defense mechanisms (DMRS variables: ODF, Mature, Neurotic, Immature including Depressive and Non-depressive Immature defenses). No significant correlations between attachment security (PACS Balance scale) or avoidance (PACS Avoidance scale) and the DMRS variables were found in the early or late sessions. In the early sessions, the PACS Resistance scale was significantly related to ODF (*r*_s_ = 0.37, *p*=0.043) and negatively associated with the DMRS Depressive Immature defenses (*r*_s_ =−0.45, *p*=0.012; see Table 2). At the late phase of treatment, the PACS Resistance scale negatively correlated with the DMRS Non-depressive immature defenses (*r*_s_ =−0.42, *p*=0.027; see Supplementary Material).

**Table 2:** Spearman correlations between early PACS attachment-related characteristics and early DMRS defense mechanisms.

	1	2	3	4	5	6	7	8	9
1. PACS Balance	–								
2. PACS Avoidance	−0.03	–							
3. PACS Resistance	−0.20	−0.58^**^	–						
4. DMRS ODF	−0.14	−0.11	−0.37^*^	–					
5. DMRS Mature	−0.21	0.16	0.16	0.55^**^	–				
6. DMRS Neurotic	−0.07	−0.28	0.13	0.21	−0.60^**^	–			
7. DMRS Immature	0.17	0.29	−0.36	−0.75^**^	−0.12	−0.68^**^	–		
8. Immature: Depressive	0.28	0.21	−0.45^*^	−0.86^**^	−0.38^*^	−0.38^*^	0.79^**^	–	
9. Immature: Non-depressive	−0.21	0.15	0.08	−0.12	0.26	−0.55^**^	0.55^**^	0.06	–

PACS, Patient Attachment Coding System; DMRS, Defense Mechanism Rating Scale; ODF, Overall Defensive Functioning; Immature Defenses were subdivided into Depressive immature and Non-Depressive immature.

* *p*<0.05;

** *p*<0.01.

### Research Question 2: Patients’ Use of Defense Mechanisms Early in Treatment and Attachment-Related Characteristics Late in Treatment

In order to establish whether there was any relationship between patients’ use of defense mechanisms early in treatment and improvement in their attachment-related characteristics during treatment, we used Spearman’s rho correlations between the defense variables (DMRS scales: ODF, Mature, Neurotic, Immature) at the early phase of the treatment and attachment variables (PACS scales: Balance, Avoidance, Resistance) at the late phase of the treatment. Results showed a significant negative correlation between early DMRS Neurotic defenses and late-phase PACS Avoidance scale (*r*_s_ =−0.44, *p*=0.020) and a significant negative correlation with the PACS Resistance scale at the end phase of treatment (*r*_s_ =−0.42, *p*=0.030). Early DMRS Immature defenses were significantly and positively correlated with late-phase PACS Avoidance scale (*r*_s_ *=0*.51, *p*=0.005) and negatively with late-phase PACS Resistance scale (*r*_s_ =−0.48, *p*=0.009; see Supplementary Material).

Based on these significant relationships between DMRS defenses early in treatment and PACS scales in the late phase of treatment, we conducted linear regressions to establish whether defense use (DMRS Immature, Neurotic defenses) in the early phase predicts attachment-related characteristics (PACS Avoidance, Resistance scales) in the late phase of treatment, after controlling for early levels of attachment-related characteristics. Since the DMRS Immature defenses category can be divided into the two mutually exclusive subcategories of Depressive immature defenses and Non-depressive immature defenses, we substituted these subcategories in the regression model, rather than the less specific DMRS Immature defense category. We used stepwise regression to assess the unique contribution of Depressive and Non-depressive defenses in predicting the change in attachment-related characteristics.

As Table 3 shows, both early Depressive and Non-depressive immature defenses significantly predicted late-phase PACS Avoidance after controlling for baseline PACS Avoidance (*B*=6.47, *SE*=2.20, *t*=2.95, *p*<0.05, Δ*R*^2^=0.14; *B*=8.43, *SE*=3.70, *t*=2.38, *p*<0.05, Δ*R*^2^=0.11; respectively). Moreover, early Non-depressive immature defenses (but not Depressive immature defenses) negatively predicted PACS Resistance at the late phase of treatment, after controlling for early PACS Resistance (*B*=−18.56, *SE*=5.48, *t*=−3.38, *p*<0.01, Δ*R*^2^=0.23). Finally, early DMRS Neurotic defenses significantly predicted late-phase PACS Avoidance after controlling for early PACS Avoidance (*B*=−0.3.84, *SE*=1.81, *t*=−2.13, *p*<0.05, Δ*R*^2^=0.06). Early-phase DMRS Neurotic defenses did not predict late-phase PACS Resistance significantly after controlling for early Resistance (*B*=5.16, *SE*=2.69, *t*=1.92, *p*=ns, Δ*R*^2^=0.10).

**Table 3:** Regression models for early DMRS defense mechanisms predicting late PACS attachment-related characteristics.

Predictor variables	Coeff	*SE*	95% CI	*F*	*df*	*p*	adj*R*^2^
**Immature defenses predicting Avoidance**							
Early Avoidance	0.52^**^	0.17	(0.18, 0.87)				
Depressive defenses	5.76^*^	2.37	(0.88, 10.64)				
Non-Depressive defenses	8.78^*^	3.69	(1.16, 16.41)	8.83	(3, 27)	0.000	0.47
**Immature defenses predicting Resistance**							
Early Resistance	0.48^*^	0.17	(0.11, 0.85)				
Non-Depressive defenses	−16.69^*^	5.85	(−28.74, −4.64)	8.55	(2, 27)	0.001	0.36
**Neurotic defenses predicting Avoidance**							
Early Avoidance	0.52^**^	0.17	(0.11, 0.85)				
Neurotic defenses	−3.84^*^	1.81	(−0.39, 10.71)	7.83	(2, 27)	0.002	0.34
**Neurotic defenses predicting Resistance**							
Early Resistance	0.48^**^	0.18	(0.18, 0.87)				
Neurotic defenses	5.16	2.69	(−7.56, −0.12)	5.71	(2, 27)	0.009	0.26

* *p<* 0.05;

** *p<* 0.01.

## Discussion

This pilot study is the first to examine patients’ defense mechanisms in relation to their attachment in a clinical sample of depressed patients and also the first to use observer-rated measures for assessing both defense mechanisms and attachment. Specifically, the present study explored the role of early-phase defense mechanisms in predicting changes in attachment-related characteristics over the course of psychotherapy.

We first hypothesized that patients with higher overall defensive functioning, more Mature defenses, and less Immature defenses would be associated with more attachment security across all sessions. This first hypothesis was not supported. We found that attachment security (PACS Balance) and PACS Avoidance were not related to defenses, but PACS Resistance was positively associated with overall defensive functioning at the early phase of treatment and negatively associated with Depressive Immature defenses in the early phase. PACS Resistance was also negatively associated with Non-depressive immature defenses at the late phase of treatment.

Our second hypothesis was partly supported, in that early-phase Immature and Neurotic defense use was related to late-phase attachment-related characteristics. We found that Immature defenses, and specifically, both Depressive and Non-depressive immature defense use and Neurotic defense use, were associated with more late-phase PACS Avoidance, even after controlling for early-phase PACS Avoidance levels. Moreover, more Non-depressive defense use during the early phase of therapy predicted less PACS Resistance at the late phase, after controlling for the effect of early PACS Resistance levels.

The positive relationship between overall defensive functioning and preoccupied attachment-related characteristics at the early phase of treatment may be explained by the fact that defensive functioning is usually at its lowest, not at the beginning of psychotherapy but somewhat later in treatment, when the patient is more deeply engaged in working on difficult topics in therapy. Thus, even though attachment-related characteristics may be detected already in early sessions, defense style of the patient when dealing with stressful conditions (or topics) may only be displayed later in therapy or across several sessions. Moreover, we assessed defenses and attachment in only one session transcript from each time point. The last sessions before termination often trigger attachment-related issues and may bring up relational insecurities, which might result in bias toward lower defensive functioning and more insecure attachment characteristics than what the patient would typically display. Although this treatment trial allowed for a pilot comparison between three different psychological treatments, the variability in the number of sessions and length of therapy across the three treatment arms (an average of 21 sessions in CBT, 17 in ST, and 62 in PDT) limited the ability to interpret the temporal relationship between defenses and attachment in our study. Future studies using more sessions per treatment may more reliably assess change processes during the course of treatment.

Another explanation for the relative lack of a cross-sectional relationship between defenses and attachment-related characteristics might also be methodological. Both defense mechanisms and attachment were coded across whole therapy sessions, as they occurred, and summary scores for both constructs were used in the subsequent analyses. It is thus possible that unrelated segments were coded as defense and as attachment episodes, with relatively little overlap, manifesting in divergent results. As such, future studies implementing a more fine-grained approach focusing on identifying episodes when defense and attachment events overlap in the transcripts may more accurately reflect the association between specific defense mechanisms and attachment-related characteristics.

When interpreting the cross-sectional associations between defense use and patient attachment, it is important to also consider that our depressed sample included patients with relatively low defensive functioning and mostly insecure attachment classification (*n*=21, 70%), with half of the patients (*n*=15, 50%) classified as preoccupied. A predominance of insecure and especially preoccupied attachment in a depressed sample is to be expected, as these have been proposed to relate to psychopathology, and specifically, depression (e.g., Laczkovics et al., 2018; Ciocca et al., 2020); however, the widely varying prevalence of the three attachment styles in our sample limited a fair comparison of patients with different attachment classifications.

It is important to also note that the comparison of the results based on self-report and observer-rated methods is limited, due to the inherent differences occurring when studying phenomena at least partly outside of awareness, such as defense mechanisms and attachment. Findings obtained by self-report measures may not be directly translatable to results with observer-rated methods, such as the AAI interview and the PACS, and vice versa.

Our results imply a longitudinal relationship between immature and neurotic defense use and attachment security, in which patients who used more immature (both Depressive and Non-depressive) or neurotic defenses early in treatment displayed an increase in PACS Avoidance late in treatment, whereas patients who used more Non-depressive immature defenses early in treatment displayed a decrease in PACS Resistance by the late phase of treatment, independently of their early attachment-related characteristics. That is, in this depressed sample, which had a high prevalence of neurotic and immature defenses at the beginning of treatment, the use of these defenses was related to a reduction in characteristics related to preoccupied attachment and an increase in avoidant attachment-related characteristics over the course of treatment. Previous studies showed that insecure attachment, and especially preoccupied attachment, is associated with more vulnerability to psychopathology and especially depressive symptoms, compared to not only secure but also avoidant attachment (Cole-Detke and Kobak, 1996; Fonagy et al., 1996; Rosenstein and Horowitz, 1996; Borelli et al., 2010; Laczkovics et al., 2018). In our study, increase in avoidance and decrease in preoccupied characteristics thus might be considered as a possible proxy for improvement in attachment-related problems within insecure attachment.

The longitudinal (but not cross-sectional) findings of our pilot study support the theorized connection between defense mechanisms and adult attachment in depressed patients, as well as the few empirical findings that examined this association in non-clinical samples. These studies found that insecure attachment is typically associated with the less adaptive defense mechanisms (e.g., Prunas et al., 2019). Whereas our study did not find the expected relationship between attachment and defense variables in the same session, our findings showed that neurotic and immature defenses are related to *change* and possibly, improvement in insecure attachment over the course of treatment.

### Limitations

Observer-rated codings are a strength but may also limit generalizability outside the session. As mentioned earlier, even though observer ratings may be less biased and better able to assess processes outside of the patient’s awareness, observer ratings are limited in that they assess patient functioning in a specific context, that is, a session, which might be affected by various circumstances, including the topic of the session or the level of alliance with the therapist. In a recent meta-analysis by Spruit et al. (2020), the type of instrument used to assess attachment uniquely contributed to the explanation of variance in depression symptoms among adolescents, and studies including self-report tools reported bigger effect sizes compared to those based on interviews and observations. Although beyond the scope of the current investigation, it would be interesting to examine whether similar patterns between attachment and defenses would emerge if self-report assessments of attachment were used.

Furthermore, the PACS observer-rated coding system at the moment does not include the fourth attachment category Unresolved/disorganized (insecure) attachment. The inclusion of an additional attachment category may differentiate within the large proportion of patients currently classified as Preoccupied in our study.

Another limitation of this study is the relatively small sample size, which allowed for running correlations on the higher order defense and attachment categories, but did not allow for testing regression or mediation models on defense levels or individual defenses. The considerable differences in treatment length, especially the significantly longer psychodynamic therapies, also limit the generalizability of our results regarding temporal changes. Furthermore, we could only report initial comparisons across treatment arms. Given that some of the effect sizes across treatment modalities were large (Avoidance *η*^2^=0.158, and Resistance *η*^2^=0.154), further studies with larger sample sizes (powered to assess between-treatment effects) are warranted. Thus, this study can be seen as an exploratory pilot study, and larger-scale studies should examine the exact nature of the relationship between defense mechanisms and attachment security, testing mediation models of attachment, defenses, and psychopathology. A better understanding of the connections between insecure attachment and immature defenses with specific symptom clusters might induce clinicians to assess and intervene both on manifest symptoms and on defensive and relational styles, to help improve severe symptoms in depressed patients during the course of treatment.

Future research examining the association between adult attachment patterns and depressive symptoms should also examine further mediators and moderators. Attachment is likely best conceptualized as one etiological factor that interacts with many contextual and individual factors influencing risk for depression later in life (Cummings and Cicchetti, 1990; Rosen and Rothbaum, 1993; Belsky, 1997; De Wolff and Van Ijzendoorn, 1997; Sroufe, 2005;). As such, the association between adult attachment and depressive symptoms may be mediated by cognitive, behavioral, relational, physiological, and affective processes (e.g., emotion regulation; Malik et al., 2015). Identifying these mechanisms may offer novel targets for the treatment of depression.

Using the PACS system to study patients’ attachment in session transcripts illustrates the potential clinical relevance of applying *post hoc* observer-rated measurements within the context of a highly controlled research design, such as an RCT. These observer codings are not only relevant with regard to the research insights they provide, but also might provide a useful clinical training tool to graduate students, who are interested in learning more about the psychotherapy process and how to attune their interventions to different types of patients. Furthermore, developing simple observer-rated methods that require minimal or no training to use are warranted. These methods could provide tools for clinicians to assess their patients’ defensive and attachment-related patterns *in situ*, at any time point during treatment, which has the potential to significantly enhance case formulation and tracking treatment-related changes over time.

## Data Availability Statement

The data analyzed in this study is subject to the following licenses/restrictions: The IRB decision did not allow publishing the dataset. Requests to access these datasets should be directed to vera.bekes@yu.edu.

## Ethics Statement

The studies involving human participants were reviewed and approved by the Research Ethics Committee of the Jewish General Hospital in Montreal, Quebec, Canada (original RCT) and Yeshiva University’s IRB (WIRB), New York, NY (secondary data analysis). The patients/participants provided their written informed consent to participate in this study.

## Author Contributions

VB: original idea, study coordination, data analysis, and manuscript write-up. KD: conceptual contribution, study coordination, and manuscript write-up. DS: conceptual contribution, data analysis, and manuscript write-up. AT: conceptual contribution, coding supervision, and manuscript write-up. CS: manuscript write-up. JP: providing data from the original RCT and conceptual contribution. All authors contributed to the article and approved the submitted version.

## Conflict of Interest

The authors declare that the research was conducted in the absence of any commercial or financial relationships that could be construed as a potential conflict of interest.

## Publisher’s Note

All claims expressed in this article are solely those of the authors and do not necessarily represent those of their affiliated organizations, or those of the publisher, the editors and the reviewers. Any product that may be evaluated in this article, or claim that may be made by its manufacturer, is not guaranteed or endorsed by the publisher.

## References

[ref003] Ainsworth M. D. S. (1978). The bowlby-ainsworth attachment theory. Behav. Brain Sci. 1, 436–438.

[ref1] Bakermans-Kranenburg M. J. van Ijzendoorn M. H. (2009). The first 10,000 adult attachment interviews: distributions of adult attachment representations in clinical and non-clinical groups. Attachment Human Develop. 11, 223–263. doi: 10.1080/14616730902814762, PMID: 19455453

[ref004] Beck A. T. Steer R. A. Brown G. K. (1996). Beck depression inventory (BDI-II) (Vol. 10. p. s15327752jpa6703_13). Pearson.

[ref2] Belsky J. (1997). Variation in susceptibility to environmental influence: An evolutionary argument. Psychol. Inq. 8, 182–186. doi: 10.1207/s15327965pli0803_3

[ref3] Berney S. de Roten Y. Beretta V. Kramer U. (2014). Identifying psychotic defenses in a clinical interview. J. Clin. Psychol. 70, 428–439. doi: 10.1002/jclp.22087, PMID: 24691714

[ref4] Blatt S. J. Levy K. N. (2003). Attachment theory, psychoanalysis, personality development, and psychopathology. Psychoanal. Inq. 23, 102–150. doi: 10.1080/07351692309349028

[ref5] Borelli J. L. Crowley M. J. David D. H. Sbarra D. A. Anderson G. M. Mayes L. C. (2010). Attachment and emotion in school-aged children. Emotion 10, 475–485. doi: 10.1037/a0018490, PMID: 20677865

[ref6] Bowlby J. (1969). Attachment and Loss: Vol 1. Attachment. New York: Basic Books.

[ref002] Bowlby J. (1980). “Attachment and loss: Loss, sadness and depression,” in Attachment and Loss: Vol. 3: Loss, Sadness and Depression. London: The Hogarth press and the institute of psycho-analysis), 1–462.

[ref7] Bowlby J. (1988). A Secure Base: Parent-Child Attachment and Healthy Human Development. New York: Basic Books.

[ref8] Brennan K. Clark C. Shaver P. (1998). “Self-report measures of adult romantic attachment” in Attachment Theory and Close Relationships. eds. Simpson J. Rholes W. (New York: Guilford Press), 46–76.

[ref9] Calati R. Osmano O. De Ronchi D. Serretti A. (2010). The use of the defence style questionnaire in major depressive and panic disorders: A comprehensive meta-analysis. Psychol. Psychother. Theory Res. Pract. 83, 1–3. doi: 10.1348/147608309X464206 19671241

[ref10] Ciocca G. Rossi R. Collazzoni A. Gorea F. Vallaj B. Stratta P.. (2020). The impact of attachment styles and defense mechanisms on psychological distress in a non-clinical young adult sample: A path analysis. J. Affect. Disord. 273, 384–390. doi: 10.1016/j.jad.2020.05.014, PMID: 32560933

[ref11] Ciocca G. Collazzoni A. Limoncin E. Franchi C. Mollaioli D. Di Lorenzo G.. (2017). Defence mechanisms and attachment styles in paranoid ideation evaluated in a sample of non-clinical young adults. Riv. Psichiatr. 52, 162–167. doi: 10.1708/2737.27909, PMID: 28845865

[ref12] Cole-Detke H. Kobak R. (1996). Attachment processes in eating disorders and depression. J. Consult. Clin. Psychol. 64, 282–290. doi: 10.1037/0022-006X.64.2.282, PMID: 8871412

[ref13] Cramer P. Kelly F. D. (2010). Attachment style and Defense mechanisms in parents who abuse their children. J. Nerv. Ment. Dis. 198, 619–627. doi: 10.1097/NMD.0b013e3181ef3ee1, PMID: 20823722

[ref14] Cummings E. M. Cicchetti D. (1990). “Toward a transactional model of relations between attachment and depression,” in Attachment in the Preschool Years: Theory, Research, and Intervention. eds. Greenberg M. T. Cicchetti D. Cummings E. M. (United States: University of Chicago Press), 339–372.

[ref15] Dagan O. Facompré C. Bernard K. (2018). Adult attachment representations and depressive symptoms: A meta-analysis. J. Affect. Disord. 236, 274–290. doi: 10.1016/j.jad.2018.04.091, PMID: 29751243

[ref006] De Wolff M. S. Van Ijzendoorn M. H. (1997). Sensitivity and attachment: A meta‐analysis on parental antecedents of infant attachment. Child Dev. 68, 571–591. 9306636

[ref16] Di Giuseppe M. Perry J. C. Lucchesi M. Michelini M. Vitiello S. Piantanida A.. (2020). Preliminary reliability and validity of the DMRS-SR-30, a novel self-report measure based on the Defense mechanisms rating scales. Front. Psych. 11:870. doi: 10.3389/fpsyt.2020.00870 PMC7479239 33005160

[ref17] Duschinsky R. (2020). Cornerstones of Attachment Research. United Kingdom: Oxford University Press.

[ref18] Dykas M. J. Cassidy J. (2011). Attachment and the processing of social information across the life span: theory and evidence. Psychol. Bull. 137, 19–46. doi: 10.1037/a0021367, PMID: 21219056

[ref19] Eagle M. (2013). Attachment and Psychoanalysis. New York: Guilford Press.

[ref20] Ein-Dor T. Viglin D. Doron G. (2016). Extending the Transdiagnostic model of attachment and psychopathology. Front. Psychol. 7:484. doi: 10.3389/fpsyg.2016.00484, PMID: 27064251 PMC4814492

[ref21] Feeney J. A. (2008). “Adult romantic attachment: developments in the study of couple relationships,” in Handbook of Attachment: Theory, Research, and Clinical Applications. eds. Cassidy J. Shaver P. R. (New York: Guilford Press), 456–481.

[ref23] Fonagy P. Leigh T. Steele M. Steele H. Kennedy R. Mattoon G.. (1996). The relation of attachment status, psychiatric classification, and response to psychotherapy. J. Consult. Clin. Psychol. 64, 22–31. doi: 10.1037/0022-006X.64.1.22, PMID: 8907081

[ref24] Fonagy P. (2001). Attachment Theory and Psychoanalysis. New York: Other Press.

[ref29] Groh A. M. Fearon R. P. Bakermans-Kranenburg M. J. van Ijzendoorn M. H. Steele R. D. Roisman G. I. (2014). The significance of attachment security for children’s social competence with peers: A meta-analytic study. Attachment Human Develop. 16, 103–136. doi: 10.1080/14616734.2014.883636, PMID: 24547936 PMC4021853

[ref30] Hames J. L. Hagan C. R. Joiner T. E. (2013). Interpersonal processes in depression. Annu. Rev. Clin. Psychol. 9, 355–377. doi: 10.1146/annurev-clinpsy-050212-185553 23297787

[ref31] Hamilton M. (1960). A rating scale for depression. J. Neurol. Neurosurg. Psychiatry 23, 56–62. doi: 10.1136/jnnp.23.1.56, PMID: 14399272 PMC495331

[ref32] Høglend P. Perry J. C. (1998). Defensive functioning predicts improvement in major depressive episodes. J. Nerv. Ment. Dis. 186, 238–243. doi: 10.1097/00005053-199804000-00006, PMID: 9569892

[ref33] Holland A. S. Roisman G. I. (2010). Adult attachment security and young adults’ dating relationships over time: self-reported, observational, and physiological evidence. Dev. Psychol. 46, 552–557. doi: 10.1037/a0018542, PMID: 20210513

[ref34] Ivarsson T. Granqvist P. Gillberg C. Broberg A. G. (2010). Attachment states of mind in adolescents with obsessive–compulsive disorder and/or depressive disorders: A controlled study. Eur. Child Adolesc. Psychiatry 19, 845–853. doi: 10.1007/s00787-010-0120-x, PMID: 20607573

[ref36] Kleinbub J. R. Talia A. Palmieri A. (2020). Physiological synchronization in the clinical process: A research primer. J. Couns. Psychol. 67, 420–437. doi: 10.1037/cou0000383, PMID: 32614224

[ref37] Kobak R. Bosmans G. (2019). Attachment and psychopathology: A dynamic model of the insecure cycle. Curr. Opin. Psychol. 25, 76–80. doi: 10.1016/j.copsyc.2018.02.018, PMID: 29614483 PMC6138578

[ref38] Laczkovics C. Fonzo G. Bendixsen B. Shpigel E. Lee I. Skala K.. (2018). Defense mechanism is predicted by attachment and mediates the maladaptive influence of insecure attachment on adolescent mental health. Curr. Psychol. 39, 1388–1396. doi: 10.1007/s12144-018-9839-1

[ref39] Lakey B. Orehek E. (2011). Relational regulation theory: A new approach to explain the link between perceived social support and mental health. Psychol. Rev. 118, 482–495. doi: 10.1037/a0023477, PMID: 21534704

[ref40] Luyten P. Campbell C. Fonagy P. (2021). Rethinking the relationship between attachment and personality disorder. Curr. Opin. Psychol. 37, 109–113. doi: 10.1016/j.copsyc.2020.11.003, PMID: 33385979

[ref41] Lyons-Ruth K. (2003). Dissociation and the parent-infant dialogue: A longitudinal perspective From attachment research. J. Am. Psychoanal. Assoc. 51, 883–911. doi: 10.1177/00030651030510031501, PMID: 14596565

[ref42] Main M. Goldwyn R. Hesse E. (2002). Classification and Scoring Systems for the Adult Attachment Interview. Berkeley, CA: University of California.

[ref43] Main M. Kaplan N. Cassidy J. (1985). Security in infancy, childhood, and adulthood: A move to the level of representation. Monogr. Soc. Res. Child Dev. 50:66. doi: 10.2307/3333827

[ref44] Malik S. Wells A. Wittkowski A. (2015). Emotion regulation as a mediator in the relationship between attachment and depressive symptomatology: A systematic review. J. Affect. Disord. 172, 428–444. doi: 10.1016/j.jad.2014.10.007, PMID: 25451448

[ref45] Martin-Joy J. S. Malone J. C. Cui X.-J. Johansen P.-Ø. Hill K. P. Rahman M. O.. (2017). Development of adaptive coping From mid to late life: A 70-year longitudinal study of Defense maturity and its psychosocial correlates. J. Nerv. Ment. Dis. 205, 685–691. doi: 10.1097/NMD.0000000000000711, PMID: 28682982

[ref49] Miller-Bottome M. Talia A. Safran J. D. Muran J. C. (2018). Resolving alliance ruptures from an attachment-informed perspective. Psychoanal. Psychol. 35, 175–183. doi: 10.1037/pap0000152, PMID: 29651196 PMC5891160

[ref50] Mullen L. S. Blanco C. Vaughan S. C. Vaughan R. Roose S. P. (1999). Defense mechanisms and personality in depression. Depress. Anxiety 10, 168–174. doi: 10.1002/(SICI)1520-6394(1999)10:4<168::AID-DA5>3.0.CO;2-5, PMID: 10690578

[ref51] Perry J. C. (1990). Defense Mechanism Rating Scales (DMRS). 5th *Edn*. Cambridge, MA: Author.

[ref52] Perry J. C. Banon E. Bond M. (2020). Change in defense mechanisms and depression in a pilot study of antidepressive medications plus 20 sessions of psychotherapy for recurrent major depression. J. Nerv. Ment. Dis. 208, 261–268. doi: 10.1097/NMD.0000000000001112, PMID: 32221178

[ref53] Perry J. C. Bond M. (2012). Change in defense mechanisms during long-term dynamic psychotherapy and five-year outcome. Am. J. Psychiatr. 169, 916–925. doi: 10.1176/appi.ajp.2012.11091403 22885667

[ref001] Perry J. C. (2014). Anomalies and specific functions in the clinical identification of defense mechanisms. J. Clin. Psychol. 70, 406–418. 24677150 10.1002/jclp.22085

[ref54] Perry J. C. Bond M. (2017). Addressing defenses in psychotherapy to improve adaptation. Psychoanal. Inq. 37, 153–166. doi: 10.1080/07351690.2017.1285185

[ref55] Perry J. C. Bond M. Diep D. (2021). Change in defense mechanisms and depression in a pilot study of antidepressive medications plus up to 18 months of psychotherapy for recurrent major depression. J. Nerv. Ment. Dis. 208, 261–268. doi: 10.1097/nmd.0000000000001112 32221178

[ref56] Perry J. C. Henry M. (2004). Studying defense mechanisms in psychotherapy using the Defense mechanism rating scales. Defense Mech. Theor. Res. Clin. Perspec. 136, 165–186. doi: 10.1016/S0166-4115(04)80034-7

[ref57] Prunas A. Di Pierro R. Huemer J. Tagini A. (2019). Defense mechanisms, remembered parental caregiving, and adult attachment style. Psychoanal. Psychol. 36, 64–72. doi: 10.1037/pap0000158

[ref58] Roisman G. I. (2006). The role of adult attachment security in non-romantic, non-attachment-related first interactions between same-sex strangers. Attachment Human Develop. 8, 341–352. doi: 10.1080/14616730601048217, PMID: 17178612

[ref59] Rosen K. S. Rothbaum F. (1993). Quality of parental caregiving and security of attachment. Dev. Psychol. 29, 358–367. doi: 10.1037/0012-1649.29.2.358

[ref60] Rosenstein D. S. Horowitz H. A. (1996). Adolescent attachment and psychopathology. J. Consult. Clin. Psychol. 64, 244–253. doi: 10.1037/0022-006X.64.2.244, PMID: 8871408

[ref61] Spruit A. Goos L. Weenink N. Rodenburg R. Niemeyer H. Stams G. J.. (2020). The relation between attachment and depression in children and adolescents: A multilevel meta-analysis. Clin. Child. Fam. Psychol. Rev. 23, 54–69. doi: 10.1007/s10567-019-00299-9, PMID: 31392452 PMC7000490

[ref62] Sroufe L. A. (2005). Attachment and development: A prospective, longitudinal study from birth to adulthood. Attachment Human Develop. 7, 349–367. doi: 10.1080/14616730500365928, PMID: 16332580

[ref63] Storch E. A. Roberti J. W. Roth D. A. (2004). Factor structure, concurrent validity, and internal consistency of the beck depression inventory?Second edition in a sample of college students. Depress. Anxiety 19, 187–189. doi: 10.1002/da.20002, PMID: 15129421

[ref64] Strauss B. Altmann U. Singh S. Schurig S. Kirchmann H. (2015). The "attachment elephant"- Are different measures of adult attachment related to the same construct? First results of a collaborative study, in *Vortrag, 46th Annual Meeting of the Society for Psychotherapy Research*: June 26, 2015.

[ref65] Talia A. Daniel S. I. Miller-Bottome M. Brambilla D. Miccoli D. Safran J. D.. (2014). AAI predicts patients’ in-session interpersonal behavior and discourse: A “move to the level of the relation” for attachment-informed psychotherapy research. Attachment Human Develop. 16, 192–209. doi: 10.1080/14616734.2013.859161 24329043

[ref66] Talia A. Miller-Bottome M. Daniel S. I. F. (2017). Assessing attachment in psychotherapy: validation of the patient attachment coding system (PACS). 1205 Clin. Psychol. Psychother. 24, 149–161. doi: 10.1002/cpp.1990, PMID: 26596847

[ref67] Talia A. Miller-Bottome M. Katznelson H. Pedersen S. H. Steele H. Schröder P.. (2019a). Mentalizing in the presence of another: measuring reflective functioning and attachment in the therapy process. Psychother. Res. 29, 652–665. doi: 10.1080/10503307.2017.1417651, PMID: 29298602 PMC6102086

[ref68] Talia A. Miller-Bottome M. Wyner R. Lilliengren P. Bate J. (2019b). Patients’ adult attachment interview classification and their experience of the therapeutic relationship: are they associated? Research in psychotherapy: psychopathology. Process and Outcome 22:361. doi: 10.4081/ripppo.2019.361 PMC7451326 32913798

[ref69] Trower P. Chadwick P. (1995). Pathways to defense of the self: A theory of two types of paranoia. Clin. Psychol. Sci. Pract. 2, 263–278.

[ref70] Vaillant G. E. (2020). “Defense Mechanisms” in Encyclopedia of Personality and Individual Differences. eds. Zeigler-Hill V. Shackelford T. K. (New York: Springer International Publishing), 1024–1033.

[ref71] Williams N. L. Riskind J. H. (2004). Cognitive vulnerability and attachment. J. Cogn. Psychother. 18, 3–6. doi: 10.1891/jcop.18.1.3.28052

