# Supplementary material for: The Relationship Between Defense Mechanisms and Attachment as Measured by Observer-Rated Methods in a Sample of Depressed Patients: A Pilot Study
Source: Front Psychol. 2021 Sep 27;12:648503. doi: 10.3389/fpsyg.2021.648503 (PMC8503562; doi:10.3389/fpsyg.2021.648503)
Supplement: Supplementary file 1 [file Table_1.docx]

**Supplemental Material**

**Table 1**

*Spearman Correlations between Late PACS Attachment-related characteristics and Late DMRS Defense Mechanisms*

**1 2 3 4 5 6 7 8 9**

1. PACS Balance —

2. PACS Avoidance .23 —

3. PACS Resistance -.25 -.65** —

4. DMRS ODF -.02 .03 -.07 —

5. DMRS Mature -.04 -.04 -.11 .54** —

6. DMRS Neurotic -.09 -.07 .25 .32 -.49** —

7. DMRS Immature .11 .14 -.16 -.77** -.10 -.77** —

8. Immature: Depressive -.07 .08 -.01 -.75** -.10 -.64** .85** —

9. Immature: Non-depressive .30 .28 -.42 -.36 -.11 -.47* .55** .13 —

*Note.* PACS = Patient Attachment Coding System; DMRS = Defense Mechanism Rating Scale; ODF = Overall Defensive Functioning; Immature Defenses were subdivided into Depressive immature and Non-Depressive immature. **p < .05; ** p < 0.01*

**Table 2**

*Spearman Correlations between Early DMRS Defense Mechanisms and Late PACS Attachment-related characteristics*

**1 2 3 4 5 6 7 8 9**

1. DMRS ODF —

2. DMRS Mature .55** —

3. DMRS Neurotic .21 -.60** —

4. DMRS Immature -.75** -.12 -.68** —

5. Immature: Depressive -.86** -.38* -.38* .79** —

6. Immature: Non-depressive -.12 .26 -.55** .55** .06 —

7. PACS Balance -.23 .11 -.32 .35 .34 .34 —

8. PACS Avoidance -.25 .06 -.44* .51 .33 .31 .23 —

9. PACS Resistance .26 -.05 .42* -.48** -.28 -.36 -.25 -.65** —

*Note.* PACS = Patient Attachment Coding System; DMRS = Defense Mechanism Rating Scale; ODF = Overall Defensive Functioning; Immature Defenses were subdivided into Depressive immature and Non-Depressive immature. **p < .05; ** p < 0.01*
